# Oral health and mental health in healthy adults, a topic of primary prevention and health care, empirical results from two online studies

**DOI:** 10.1007/s12144-022-04121-8

**Published:** 2023-01-07

**Authors:** Cornelia Herbert

**Affiliations:** Applied Emotion and Motivation Psychology, Institute of Psychology and Education, Ulm University, 89081 Ulm, Germany

**Keywords:** Oral health, Mental health, Depressive symptoms, Eating disorders, Health behavior, Perceived stress

## Abstract

Oral health is an important, yet often neglected aspect of health and well-being. Among geriatric populations, oral diseases, poor oral health, and reduced oral hygiene behavior are major comorbid factors of geriatric diseases such as dementia, cardiovascular diseases, or mental health conditions. However, little is known so far about the relationships between oral health and mental health in the younger adult population, who is yet not suffering from any disorder. In the present manuscript the results of two online studies, online study 1, *n* = 133, all-female sample and online study 2: *n* = 29, mixed gender sample, are reported that investigate the relationship between self-reported oral health impairments and preclinical symptoms of mental disorders including self-reported symptoms associated with depression or eating disorders as well as self-reported perceived stress and behavior change related to the COVID-19 pandemic. The results of both studies support links between self-reported oral health impairments, self-reported depressive symptoms (including anxiety and current affect) and symptoms related to eating disorders (such as bulimic behavior, body dissatisfaction or drive for thinness) among young adults and suggest that perceived stress may contribute to both, impaired oral health and impaired mental health in young adults (women and men).

**Supplementary Information:**

The online version contains supplementary material available at 10.1007/s12144-022-04121-8.

## Introduction

Oral health is an important, yet often neglected aspect of general health and well-being among people worldwide suffering from oral health impairments (e.g., D’Souza et al., 2022; Dye, 2017; Jin et al., 2016; Petersen et al., 2005). In addition, mental health impairments such as depression or anxiety have increased substantially worldwide (Kessler et al., 2015). Among the elderly and geriatric populations, there is plenty of proof in the scientific literature for a relationship between oral health, mental health and general health (e.g., Brennan & Strauss, 2014; Cademrtori et al., 2018; Chávez & Ship, 2000; Coll et al., 2020; Ghezzi & Ship, 2000;  Gil-Montoya et al., 2015). In particular it has been suggested that oral diseases, poor oral health and reduced oral hygiene behavior are major comorbid factors of geriatric diseases such as dementia, cardiovascular diseases, or mental health impairments such as depression, and therefore in the elderly significantly contributing to cognitive, mental and physical decline (e.g., Brennan & Strauss, 2014; Cademartori et al., 2018; Chávez & Ship, 2000; Coll et al., 2020; Ghezzi & Ship, 2000; Gil-Montoya et al., 2015). In addition, immobility (hospitalization) but also physical inactivity and sedentarism have been discussed in the scientific literature as negative accelerators promoting the decline of oral, mental and physical health in these population groups (Terezakis et al., 2011). While significant evidence about the relevance of the relationship between oral health, physical and mental health and well-being has already been accumulated across studies in the elderly, research such as survey studies investigating these relationships in the younger adult population have started only recently (Tiwari et al., 2021), and this, although, as will be outlined in detail below, oral health can be seriously affected in adults as well, especially in vulnerable target groups suffering from psychosomatic disorders such as cardiovascular disorders or mental disorders, such as major depressive disorders (MDD) or eating disorders (EDs).

As far as the prevalence of mental disorders is concerned, MDD is among the most frequent mental health conditions worldwide with an ever increasing earlier onset during the lifespan; first episodes of mood disorders are expected to appear at the age of 20.5 years (e.g., Solmi et al., 2022; Polanczyk et al., 2015). Besides a genetic and biological predisposition, traumatic life experiences, sociodemographic variables such as poor economic and educational status are known to accumulate the life time risk of MDD (e.g., Kessler et al., 1994). The risk of depressive symptoms has become a public health burden ranked to be the second major cause of disability worldwide (Liu et al., 2020). Eating disorders (EDs) such as anorexia nervosa, bulimia or binge-eating disorder are among the common chronic mental disorders in adults in developed countries. Eating disorders have an early onset during puberty, with often a higher vulnerability in girls and women than boys and men (Striegel-Moore et al., 2009). Eating disorders show later chronification during adulthood, especially if untreated. Notably, dissatisfaction with the own body and drive for thinness as the societal norms of a lean body in women or a muscular body in men, and the internalization of those norms as ideal body images are the major risk factors of developing eating disorders, besides genetic, biological, social or psychological factors (e.g., dietary restraint, family history of an eating disturbance, etc.), Hilbert et al., 2014. Moreover, among the mental health conditions, eating disorders have the highest mortality and morbidity (Hilbert et al., 2014).

There is evidence for a relationship between oral health and MDD or EDs in adults. Concerning eating disorders (EDs), either food restriction and fastening, or extreme dieting or periods of dieting, overeating and unhealthy eating in conjunct with self-induced vomiting, misuse of laxanthia, and preference for acid food, have been suggested as the major factors that could explain the direct relationship between oral health and EDs (e.g., Frydrych et al., 2005; Kisely et al., 2015). Especially, dental erosion is one of the most often found oral symptoms in patients diagnosed with e.g., bulimia nervosa due to vomiting and extreme food choices (Kisely et al., 2015). Moreover, malnutrition and restricted eating in anorexia nervosa can contribute to diseases of gum, teeth and mouth, the latter also having effects on social life of the patients including emotional distress and reduced social interaction (Kisely et al., 2015). Dental and oral health problems, and oral diseases of the gum such as periodontitis are also evident in people suffering from depressive symptoms (e.g., Cademartori et al., 2018; Kisely et al., 2016). Changes in behavior and in subjective experience related to depressive symptoms in MDD might facilitate risk factors that contribute to wrong dental care, poor dental health and damage of the oral cavity such as smoking, alcohol consumption, or wrong dieting and eating behavior (increased sugar and fat consumption). Thus, poor oral health in MDD might occur during or secondary to the disorder as a consequence of medication intake, symptom-related loss of interest or motivation making it difficult for these patients to adhere to a structured and healthy lifestyle including oral hygiene and regular dental visits (Alkan et al., 2015; Kisely, 2016). These changes in behavior might not be specific risk factors contributing only to poor oral health. These factors promote the risk of non-communicable diseases (NCDs), to which oral diseases are linked. Life-style related risk factors may improve or further impact the severity and risk of mental disorders and oral diseases (Walsh, 2011). Thus, from a psychological point of view, one could speculate that the relationship between oral health and mental health is multifactorial and sharing joint mechanisms. Negative or positive interactions between these factors during adolescence and adulthood could then pave the way for good or poor oral and mental health later in life. Psychological stress on the one hand and people’s health behavior and people’s sensitivity to physical, sensory and bodily changes on the other hand might be important factors modulating the risk of poor or good oral and mental health (e.g., Jin et al., 2016). 

### Aim of the present studies and hypotheses

Despite the evidence outlined above that oral health and mental health are linked in vulnerable target groups suffering from mental health conditions or oral health impairments, several open questions remain: First, does the relationship between poor oral health and poor mental health exist prior to the diagnosis of oral, mental or physical ill-health? In other words, will pre-clinical self-reported symptoms of mental disorders associated with depression or eating disorders show relationships with oral health in healthy adults as well? Second, if so, what could be common mechanisms of these relationships? Could perceived stress, lifestyle related factors, anxiety, mood and affect be contributing factors? In this manuscript, results from two online studies will be reported that aimed to give answers to these questions. The online study 1 investigates the relationship between risk of eating disorders (EDs) and depressive symptoms, and self-reported oral health in women, all healthy adults with no history or clinical diagnosis of psychiatric or neurological diseases including EDs or depression, respectively. In addition, the relationship between oral health and the participants’ health behavior as well as their ability to sense changes in their body (such as sensations in their mouth) is investigated. The online study 2 investigates the hypothesis of a relationship between perceived stress, oral health and severity of self-reported depressive symptoms. To this end, a smaller sample of healthy women and men at the same age of the participants of the online study 1 and who akin to the participants of study 1 report no history of mental, psychiatric or neurological disorders is investigated. First, the occurrence and the severity of self-reported symptoms of depression or the risk of EDs will be assessed and analysed in conjunct with the occurrence of oral impairments to better understand the relationship between self-reported mental and oral health impairments in healthy adults who at the time of study participation are yet without a clinical diagnosis of depression or an eating disorder (ED). Next, the mean scores of the study samples for depressive symptoms, eating disorders (EDs) and self-reported oral impairments will be compared with norms of reference populations taken from the literature. Next, correlations will be assessed between self-reported symptoms of depression, eating disorders (EDs) and oral health impairments. Therefore, the psychological assessment includes standardized self-report measures for the evaluation of the risk of depressive symptoms (online study 1 and online study 2) and the risk of eating disorders (online study 1) and of oral health and associated oral health impairments (online study 1 and online study 2). Oral health and related impairments are assessed with the Oral Health Impact Profile (OHIP, German version: John et al., 2002). The OHIP is a questionnaire measuring self-reported oral dysfunction, discomfort and disability common to most oral disorders or oral syndromes (John et al., 2002). The seven dimensions/subscales of the OHIP capture all facets of the multidimensional concept of oral health according to the general definition of oral health as defined by the World Dental Federation as “health of the mouth” and “ability to speak, smile, smell, taste, touch, chew, swallow and convey a range of emotions through facial expressions with confidence and without pain, discomfort and disease of the craniofacial complex (head, face, and oral cavity)”, cited from the World Dental Federation (see https://www.fdiworlddental.org/). In addition, health behavior (online study 1), self-reported sensitivity to bodily symptoms as well as self-reported perceived stress (online study 2), anxiety (online study 1), mood and affect (online study 1 and 2) will be assessed by means of standardized self-report questionnaires and survey items (for details see Methods). In line with the aim of the studies, the following hypotheses will be investigated:

#### Oral health and depressive symptoms

The severity of self-reported depressive symptoms is expected to positively correlate with self-reported oral health impairments. It is hypothesized that this relationship should be stronger in the subsample of participants scoring above the cut off scores distinguishing those participants reporting no depressive symptoms from those participants reporting minimal, minor, moderate or severe depressive symptoms.

#### Oral health and risk of eating disorders (EDs)

A significant positive correlation is expected between self-reported oral health impairments and self-reported symptoms central to eating disorders (EDs). Correlations between symptoms of EDs and oral health are expected to comprise all seven domains of oral health impairments captured by the OHIP questionnaire (John et al., 2002). If oral health impairments occur in conjunct with the risk of EDs, it is expected that participants who score above the cut off scores for the risk of EDs will show higher mean OHIP scores than participants scoring below the questionnaire’s cut off scores.

#### Oral health, depressive symptoms and eating disorder symptoms (EDs)

In addition, the relationship between oral health, depression and eating disorders (EDs) is further explored by examining whether self-reported impairments in oral health are more pronounced in those participants reporting symptoms related to eating disorders or depressive symptoms compared to the participants who are reporting no symptoms. In addition, it is investigated if the participants who report no or little impairment in oral health and answer all questions of the OHIP with “no” or “almost never” differ in self-reported symptoms of EDs or depression from those participants who report oral health problems in the OHIP by answering the items with "fairly often" or "very often”.

#### Oral health, health behavior and sensitivity to bodily symptoms

To explore whether oral health can be positively or negatively influenced by the participants’ health behavior as well as their sensitivity towards bodily symptoms, correlations between the participants’ self-reported health behavior (i.e., people's actions they take for disease prevention and health promotion), and their awareness of bodily changes and their self-reported oral health impairments are explored. The hypothesis is tested that self-reported health behavior and the perception, awareness or sensitivity for these changes are positively or negatively correlated with self-reported oral health impairments.

#### Oral health, depressive symptoms, anxiety, affect and perceived stress

State and trait anxiety and current positive and negative affect are important comorbid factors in the genesis of mental and oral health complaints and depressive symptoms and anxiety might be related, even prior to the manifestation of clinically relevant symptoms. Moreover, affect and mood including state and trait anxiety might play a role in impairing oral health and increasing the risk of oral diseases, e.g., by influencing symptom perception, awareness of bodily symptoms or health behavior. Therefore, the hypothesis will be explored that in healthy adults, trait and state anxiety and positive or negative affect (as felt right in the moment) correlate with self-reported oral health complaints, in addition to depression or eating disorder symptoms. Moreover, it is tested if self-reported perceived stress (assessed in the online study 2) correlates with self-reported impairments in oral health.

#### COVID-19 and oral health, depressive symptoms, risk of EDs and perceived stress

The online studies were conducted during the COVID-19 pandemic, shortly after the pandemic outbreak in Germany and during the time period of the first lockdown in Germany during May 2021 – August 2021. Previous survey studies have shown that the pandemic situation and its necessary restrictions in social, public and work life posed a huge threat to the whole population in 2020 (e.g., Iranmanesh et al., 2021; Talevi et al., 2020), requiring rapid behavior change in nearly all domains of life (social, public, or work). This has facilitated the risks of mental disorders considerably and probably those of oral health as well (e.g., Iranmanesh et al., 2021; Talevi et al., 2020). To explore the immediate effects of pandemic-related changes in behavior, mental and physical health, including changes in oral health, a short self-designed COVID-19 survey was included in the online study 1 as well as in the online study 2 asking for changes in affect, physical health and lifestyle related behavior caused by the pandemic situation. This allows the control of pandemic-related changes that could contribute to an increase in self-reported changes in depressive symptoms, dissatisfaction with the body related to eating disorders and bodily and oral sensitivity, oral perception and oral problems assessed with the German version of the OHIP questionnaire (John et al., 2002).

## Methods

### Participants, study design and questionnaires

In total, *N* = 336 volunteers followed the invitation to the online study 1. Of these, *n* = 184 gave written informed consent prior to the participation of the study. From this sample, *n* = 8 volunteers dropped out after giving informed consent, *n* = 2 dropped out after filling in the sociodemographic items, whereas *n* = 37 dropped out while filling out the questionnaires. The data of *n* = 4 participants were lost due to technical problems of the survey software. Thus, *N* = 133 participants filled in the full survey and these participants constitute the final study sample of the online study 1. In the online study 2, *N* = 41 women and men followed the invitation of the online study. Of these, *n* = 6 dropped out immediately, *n* = 3 after having given written informed consent. *N* = 29 women and men filled in the full questionnaire and these participants are constituting the final study sample of the online study 2. Inclusion criteria for study participation were in both studies an age of 18 years and older, age range 18–65 years, no history of mental disorders such as depression or eating disorders (EDs) or receiving medication for any of these. After giving written informed consent, the participants filled in a number of sociodemographic questions including in both studies questions about age, education, and native language (German native or first language, other languages), highest education including pursued academic career or job. In addition, they were asked about current neurological, cardiovascular or somatic diseases or sensory handicaps (including vision, hearing, or reading), current medication and regular consumption of drugs, alcohol, or tobacco/nicotine. The participants who were taking part in the online study 1 were additionally asked about their height and weight for estimation of the body mass index (BMI). Severity of depressive symptoms was, in both online studies, assessed with the German version of the Beck Depression Inventory II (BDI-II, German version: Hautzinger 2010). The Beck Depression Inventory asks for the continuous presence of depressive symptoms in a time window of the last two weeks. It contains 21 items that can be answered by choosing from options ranging from never to always and offers cut off ranges for the distinction between “no depressive symptoms” (0–8), mild (9–13), moderate (14–19) to severe depressive symptoms (20–28) or major depressive symptoms (29–63). The BDI-II was used in both online studies (study 1 and study 2) for the categorization of the participants into those with different severity of depressive symptoms including those participants without self-reported depressive symptoms. Due to the online character of the studies, the item for suicidal ideation was not included in the BDI-II. Risk of eating disorders (EDs, online study 1) was assessed with the German version of the Eating Disorder Inventory (EDI-2, German version: Thiel & Paul, 2006). Of the EDI-2, only the three subscales, dissatisfaction with the body, bulimia, and drive for thinness were included, because these three scales have proven of high diagnostic relevance for the estimation of the risk of EDs including anorexia nervosa, bulimia nervosa or restrained eating (Thiel & Paul, 2006). The EDI-2 has norms for each subscale for women (and men) diagnosed with EDs (e.g., anorexia nervosa or bulimia nervosa). The norms of these clinical samples were used for the calculation of cut off scores for the categorization of the participants (women, in study 1) into those with and without risk of EDs (Thiel & Paul, 2006). The participants’ positive and negative affect (online study 1 and online study 2) as well as their state and trait anxiety (online study 1) were assessed with the PANAS (Positive Affect Negative Affect Scales (German version: Breyer & Bluemke, 2016)) and the Spielberger State and Trait Anxiety Inventory (STAI, Spielberger, 1983; German version: Laux et al., 1981). The STAI offers norms and cut off scores to distinguish clinical from non-clinical anxiety. Cut off scores distinguishing clinical for clinical groups regarding trait and state anxiety are available from the literature (e.g., Julian, 2011). Health behavior was assessed with survey items in accordance with the assessment of multiple health behavior. Awareness of bodily symptoms was assessed with the PBC, Private Body Consciousness Scale (Miller et al., 1981; German version: own translation) with a particular focus on only five items of the PBC questionnaire examining on a seven-point Likert scale from 1 (not at all) to 7 (at all), the awareness of bodily symptoms. The items included the perception of fluctuations in body temperature; the perception of stomache and hunger; the beating of the heart, dryness of mouth and throat and sensitivity to inner tensions of the body. Perceived stress was assessed in the online study 2 with the German version of the Stress-Coping-Inventory (SCI, German version: Satow, 2012). The SCI contains 7 subscales that are capturing stress evoked by major psychological domains, highly relevant to the younger adult population, including stress due to uncertainty (job, social, private relationship), excessive demands (work load), or loss (death, illness (self-family, friends), respectively. Moreover, the SCI allows the assessment of positive and negative stress coping strategies and of perceived psychophysiological stress symptoms. To specifically assess stress-related perceived changes in oral health and daily stress, additionally four single survey items were designed asking on a 4-point Likert scale whether the participants had noticed stress-related changes in the past months regarding teeth, mouth or dentures and feelings of impairments from the experience of daily stress (Likert scale: 1–7). Oral health was assessed with the German version of the OHIP oral health impact profile (John et al., 2002). The OHIP is available in different validated version (short 14 items version and long, 49 item version). The OHIP-14 was used in the online study 1. The OHIP-49 was used in the online study 2. The short OHIP-14 as well as the long OHIP 49 questionnaires comprise the same 7 subscales thereby allowing full assessment of the impairments related to all dimensions of oral health. The sum scores of the seven subscales of both versions of the OHIP allow to quantify the degree of physical disability, physical pain (e.g., sensitivity of teeth), and functional limitation (e.g., difficulty chewing) due to oral problems, in addition to impairments arising from psychological problems and social disability (cognitive impairments such as reduced concentration; social life: avoiding social interaction), handicap (e.g., being unable to work productively), and psychological discomfort due to negative changes in oral health. In addition, a total score of the OHIP can be calculated that allows for quantification of the general impairment in oral health occurring within a time period of the last month. Both versions of the OHIP have norms from dental patients with functional oral disabilities or oral disorders (see John et al., 2002). These norms were used as a reference for the estimation of the severity of oral health impairments reported by the participants of the online study 1 and the online study 2. Finally, the online study 1 as well as the online study 2 contained Covid-19 pandemic related questions asking for specific changes in emotion or affect, mood, and health behavior (eating, drinking, or exercise) elicited by the pandemic situation. The COVID-19 survey items could be answered by choosing “yes” or “no” answers, by answering a Likert scale or by providing changes in emotion and physiological arousal on the non-verbal Self-Assessment Manikin Scale (SAM, Bradley & Lang, 1994) for Covid-19 related mood assessment. Table 1A (see supplement) gives an overview of the sociodemographic questions, the standardized questionnaires and the pandemic items assessed in the two online studies. The online study 1 as well as the online study 2 were conducted with Lime Survey Professional software (https://www.limesurvey.org/de/) using a version of Lime survey that allows the storage of data on the university’s server. Both online studies were advertised via email groups and social networks. The online design of study 1 is part of a larger project of the author with additional questionnaire data, experimental measurements and longitudinal assessment of eating disorders and depressive symptoms which was approved by the local ethics committee. Therefore, no separate or additional ethics approval was included for only the online part reported in this manuscript.

The online studies are basic research. The participants were debriefed about the purpose of the study. The study participation was voluntary, participants could opt out the study at any time of the study without undue request, they were debriefed about the inclusion and exclusion criteria before the start of the study, the data was collected anonymously and treated according to data privacy policies. The volunteers had to give written informed consent prior to study participation.

### Data analysis

Sociodemographic data, prevalence of depressive symptoms, eating disorders (EDs), trait and state anxiety, positive and negative affect, perceived stress and impairments of oral health as well as the COVID-19 related items are reported descriptively for both studies (online study 1 and online study 2). Sum scores, group means including standard deviations (SDs), percentage scores (%), and prevalence of symptoms including depression, eating disorders (EDs), and oral health impairments are based on cut off scores from reference samples taken from the literature (see Results). The relationships between oral health impairments, depressive symptoms, eating disorders (EDs), health behavior, body perception/awareness, anxiety, affect and perceived stress are statistically tested by means of correlation analysis where appropriate. In addition, in the online study 2, correlation analyses are performed between self-reported perceived stress symptoms and self-reported oral health impairments. Moreover, in the online study 1, the risk of depressive symptoms and the risk of eating disorders (EDs) are statistically compared between the group of participants reporting no oral health related symptoms in the OHIP questionnaire and the group of participants reporting oral health impairments in the OHIP questionnaire. Furthermore, it is tested if the group of participants at risk of depression or at risk of eating disorders (EDs) significantly differ in the oral health domains assessed with the OHIP questionnaire from the participants reporting no risk of EDs or depressive symptoms. Repeated measures or univariate measures of variance (ANOVAs) were used for statistical testing and significance is reported for *p* < 0.05, uncorrected.

## Results

Online Study 1 (all-female sample)

### Sociodemographics

The participants of the online study 1 had a mean age of 25 years, *SD* = 8.0. The age of the participants ranged from 18 years – 63 years. Although an age above 30 years was not an exclusion criterion, 90.2% of the women in the final sample were between 18–29 years old, in line with the study’s aim to reach especially younger adults. The study sample was relatively homogenous in terms of sociodemographic variables: 89.5% of the participants had a high school degree, 36.8% pursued an academic career, and 8.3% had an apprenticeship. 24.8% of the participants reported intake of types of medication (e.g., L-Thyroxin) that were no exclusion criterion. All participants reported to speak German as their first language, 10.5% of the participants were multilingual and reported to speak more than one language. All participants reported to live in Germany. Body Mass Index (BMI), calculated from height and weight, ranged between the scores of 16.04 – 39.64 and an average mean score of *M* = 21.81, *SD* = 3.68. The average BMI scores revealed that 77.4% of the women of the online study 1 were of normal weight, 11.3% were underweight and 7.5% were overweight and 3.8% were obese.

### Depressive symptoms

**Fig. 1:**
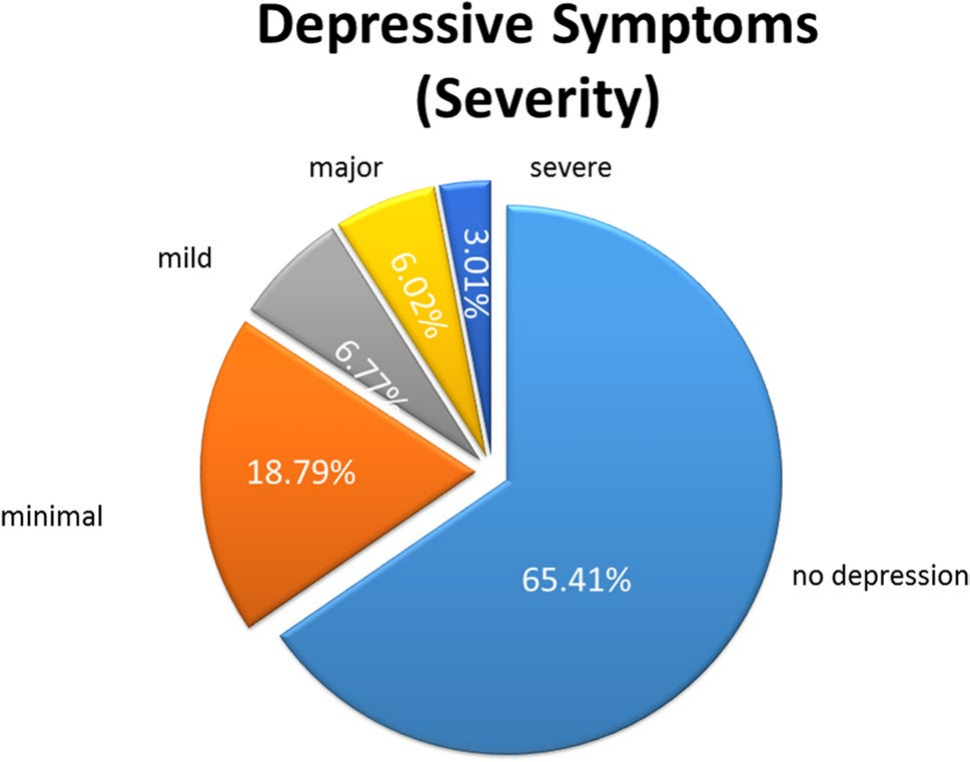
Online study 1: prevalence of self-reported depressive symptoms in % on the BDI-II according to cut off scores

The average sum score of the BDI-II of the all-female sample of the online study 1 was below the cut off score of risk of depression, *M* = 7.89, *SD* = 7.34. However, the BDI-II sum scores ranged from 0 – 40. Analysis of the sum scores of each participant according to the BDI-II cut off scores for classification of individuals with “no depression” (0–8), minimal depression (9–13), mild depression (14–19), major (20–28) or severe (29–63) depressive symptoms revealed the following distribution: 65.41% of the participants reported no depression, 18.79% see Fig. 1 reported minimal depression, 6.77% please see Fig. 1 of the participants reported mild depression, 6.02%, please see Fig. 1 below reported major and 3.01% reported severe depressive symptoms. The results are illustrated in Fig. 1. In addition, in non-clinical samples, a cut off score of 14 in the BDI-II is used to differentiate between individuals with no depression (< 14) and individuals with subclinical depression (Glischinski et al., 2021). 15.8% of the participants had a cut off score of 14 in the BDI-II and higher.

### Risk of eating disorders (EDs)

**Fig. 2:**
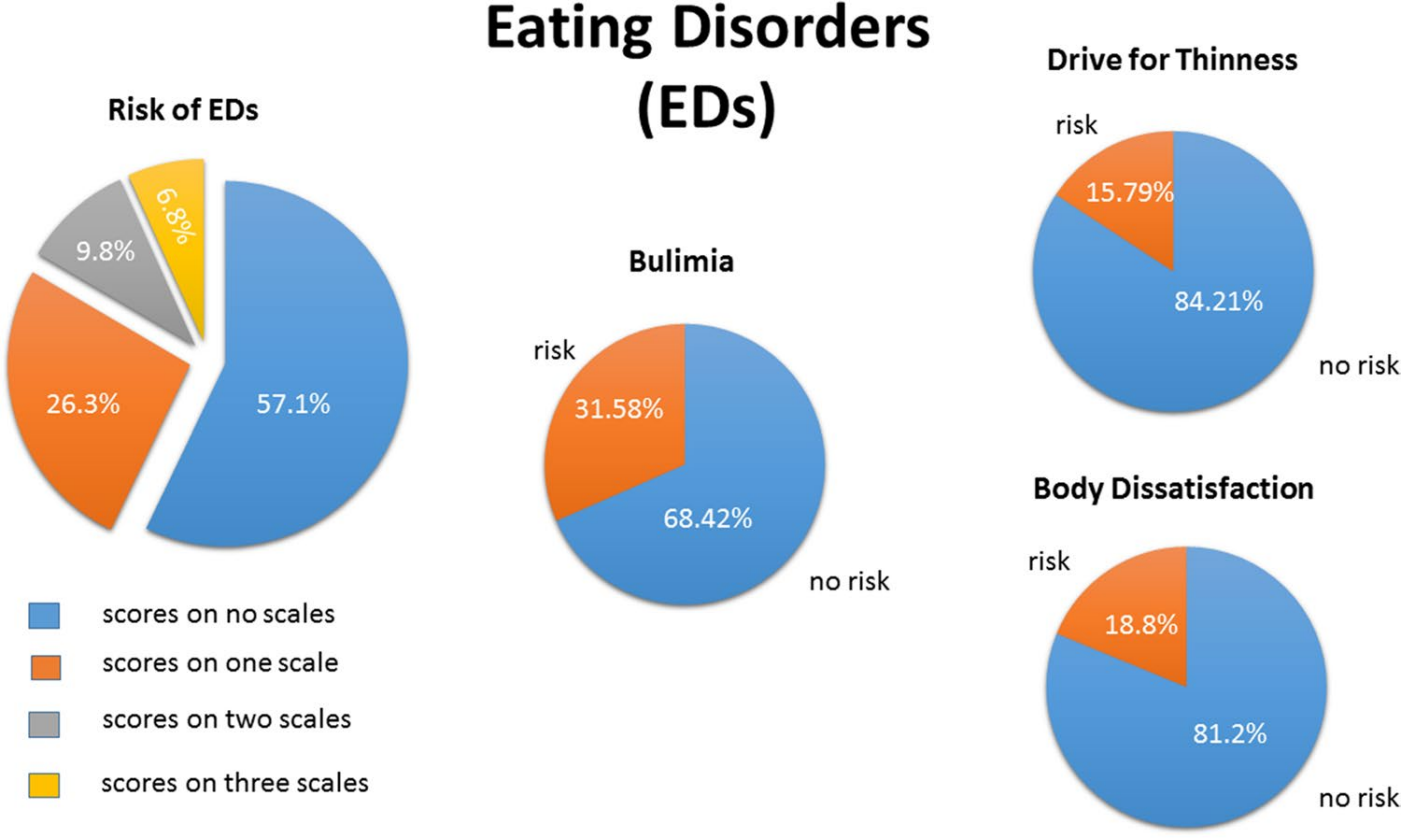
Online study 1: prevalence of self-reported eating disorder symptoms in % on the EDI-2, figure on the left, figures on the right: participants (women) in % who scored on the EDI-2 scales, bulimia, drive for thinness, or body dissatisfaction below with no risk or within (risk) the range of the clinical samples (see text for details)

Body dissatisfaction, bulimia scores and scores for drive of thinness as assessed with the EDI-2 inventory are reported in Table 2A in the supplement for the all-female sample of the online study 1. The average mean score of each of the three eating disorder subscales was *M* = 26.47, *SD* = 9.55, *M* = 12.59, *SD* = 4.70, and *M* = 17.79, *SD* = 7.84, for body dissatisfaction, bulimia and drive for thinness, respectively. Compared to the scores reported for the German reference samples of eating disorder patients in Thiel and Paul (2006), the mean scores of the three subscales of the all-female sample were below the mean ED scores of the patient reference samples. To determine women with a risk of EDs, the individual scores of each of the participants on each of the three subscales of the EDI-2 was compared with the scores reported for the German reference samples in Thiel and Paul (2006). In total, as shown in Fig. 2, 57.1% of the women of the online study 1 did not score on any of the three EDI-2 scales within the scores of the reference sample of women. When each of the three scales was considered separately, 18.80% of the women, aged between 18–32 years received scores within the range of one of the female reference samples (anorexia, bulimia or binge eating) for body dissatisfaction (BDISS). 31.58% of the women, aged 18–65 years received scores within the range of the reference samples for bulimia (BUL), and 15.79% of the women aged 18–28 years received scores within the range of one of the reference samples for the scale drive for thinness (THIN). Figure 2 summarizes the percentage of women with and without risk of EDs according to the scores taken from the respective German reference samples in Thiel & Paul, 2006. In addition, 58.3% out of the women with a risk of EDs reported depression scores of 14 and higher on the BDI-II.

### Positive and negative affect, state and trait anxiety

On average, positive affect (PA) was more pronounced than negative affect in the study sample, PA: range: 13–44, *M* = 28.2, *SD* = 6.71 vs. NA: range 10–32, *M* = 13.67, *SD* = 4.71. Mean scores of state and trait anxiety ranged from 21–78 (state) or from 20–72 (trait), respectively. The mean scores of the study sample were below the cut off scores of clinical samples, *M* = 37.42, *SD* = 10.45; trait: *M* = 38.28, *SD* = 10.72. However, 36.09% (state) and 33.08% (trait) of the participants had a state or trait anxiety score at or above the cut off scores reported by clinical samples (Julian, 2011).

### Health behavior and body awareness/perception

The female participants of the online study 1 reported on average a good health behavior before and still during the COVID-19 pandemic (see Table 3A in the supplement). Consumption of alcohol and smoking ranged low. Regular doctor visits for health care prevention dropped during the COVID-19 pandemic due to lockdowns, however, attempts of stress coping and stress regulation were similarly pronounced during as compared to before the COVID-19 pandemic. The mean scores of the PBC items reflecting awareness or sensitivity of changes in bodily functions were *M* = 21.77, *SD* = 5.43, suggesting that the participants had a mean score of body awareness with scales for the 5 items taken from the subscale of the PBC ranging from 1 -7.

### Oral health (OHIP-14)

The average sum score of the OHIP-14 of the all-female sample of the online study 1 was *M* = 2.76, *SD* = 4.72. The mean sum score of the OHIP-14 of the all-female sample showed a similar distribution to the reference population reported in John et al., 2002. Of all items of the OHIP-14, the items asking for oral pain including sensitivity of the teeth, restrained eating due to these difficulties and difficulties to relax due to oral health problems were those with the highest group mean scores. In total, about 55.6% of the all-female sample answered all OHIP-items with “no” (0) or “little” (1) impairments. A summary of the mean scores of all self-report measures described above is provided in Table 2A and 3A in the supplement.

## Online Study 2 (mixed gender sample)

### Sociodemographics

The participants of the online study 2 (*n* = 18 women, *n* = 10 men, *n* = 1 diverse) had a mean age of *M* = 33.1 years, *SD* = 13.6. The age of the participants ranged from 18–63 years. 89.66% of the participants had a high school degree, 75.86% pursued an academic career. 24.14% of the participants of the online study 2 reported intake of types of medication (e.g., L-Thyroxin) that were no exclusion criterion. All participants reported to speak German. None of them reported a history of past or current mental, psychiatric or somatic disorders.

### Depressive symptoms and positive and negative affect

**Fig. 3:**
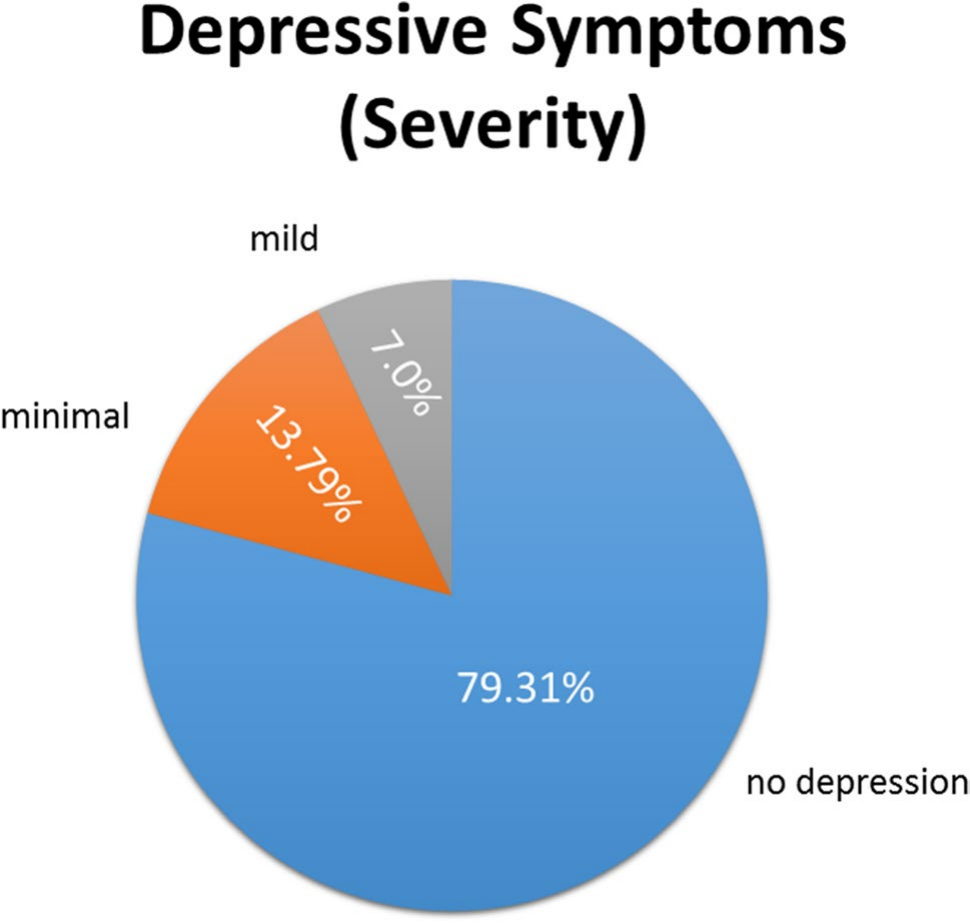
Online study 2: prevalence of self-reported depressive symptoms in % on the BDI-II according to cut off scores (no depression, minimal or mild depressive symptoms)

The average sum score of the BDI-II of the participant sample of the online study 2 was below the cut off score of risk of depression, *M* = 4.83, *SD* = 5.06. The analysis of the sum scores of each participant showed that 79.31% of the participants reported no depression, 13.79% scored in the range of minimal depression, 7.00% please see Fig. 3 had mild depression and none of the participants reported major or severe depressive symptoms. In total, 6.90% of the participants had a cut off score of 14 and higher, reaching subclinical depression. For a graphical illustration see Fig. 3. Akin to the all-female sample of the online study 1, positive affect was more pronounced than negative affect in the sample of the online study 2: positive affect (PA) ranged from 13–44, *M* = 28.2, *SD* = 6.71 vs. negative affect (NA) ranging from 10–32, *M* = 13.67, *SD* = 4.71.

### Health behavior, oral health behavior and body awareness/perception

The majority of the participants of the mixed gender sample of the online study 2 reported good health behavior (see for an overview Table 3B in the supplement). Stress coping by means of alcohol, smoking or reduced sleep was not highly pronounced. The participants reported to visit the doctors and the dentist regularly for health prevention. 75.86% reported regular dental visits and regular daily oral hygiene behavior. On average, the participants reported not to be hypo- or hypersensitive to bodily functions. On scales ranging from 0–5, most of the participants answered these survey questions by sometimes or barely.

### Perceived stress

The participants of the online study 2 reported to experience stress, *M* = 44.35, *SD* = 5.76. In addition, 17.24% of the participants, 37.93% of the participants, and 20.69% of the participants reported to feel stressed by the daily routine very often, often, or sometimes. The perceived stress (assessed with the standardized Stress Coping Inventory, SCI, (Satow, 2012) was mainly related to stress due to uncertainty and excessive demands than due to actual experiences of negative life events (e.g., job loss). In addition, the participants reported physical stress symptoms in the SCI, including headache, stomache, nightmares, sexual disinterest, etc., *M* = 21.79, *SD* = 6.47. The average mean scores for active and positive coping strategies were higher *M* = 12.59, *SD* = 2.36, than for negative stress coping strategies, *M* = 11.82, *SD* = 3.05, such as stress coping by alcohol consumption, *M* = 6.07, *SD* = 1.91.

### Oral health (OHIP-49)

**Fig. 4:**
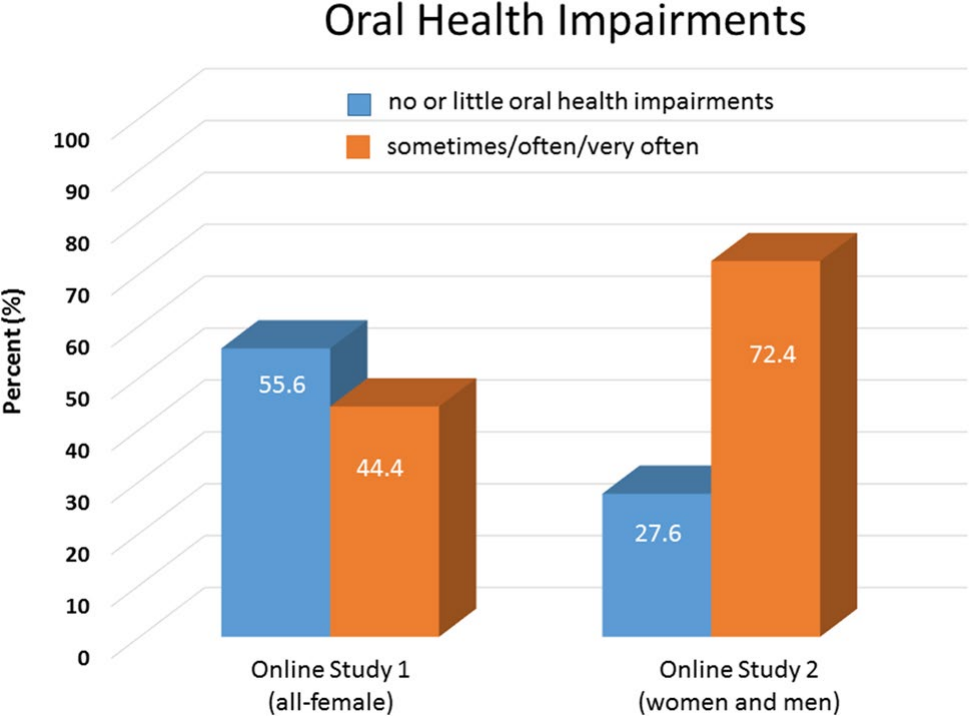
Self-reported oral health impairments in the OHIP questionnaire. Left side: results from the online study 1, right side: results from the online study 2

The average mean score of the OHIP-49 questionnaire of the participant sample of the online study 2 was *M* = 14.24, *SD* = 23.94, and thus, similarly pronounced to the healthy adult reference population reported in John et al., 2002. The mean scores of the subscales of the OHIP-49 are shown in Table 3B in the supplement. As shown in Table 3B, the subscales “functional limitation” “physical pain” and “psychological discomfort” were those with the highest group mean scores. In total, only 27.6% please see Fig. 4 of the participants of the sample of study 2 answered all OHIP-items (range: 0–4) with “no” (0) or “little” (1) impairments. The percentage of oral health impairments reported by the participants of the online study 2 (women and men) are illustrated in Fig. 4, together with the oral health impairments reported by the participants of the online study 1 (women).

### Changes due to the COVID-19 pandemic (online study 1 and online study 2)

The COVID-19 pandemic had a significant impact on self-reported changes in mood. In the online study 1, participants reported a significant decline in feelings of pleasantness, *F*(1, 76) = 17.81, *p* < 0.001, but not in emotional arousal, *F(*1, 76) = 0.544, *p* = 0.46, due to the pandemic situation compared to their feelings before the pandemic situation. 87.0% of the women reported to be considerably stressed by the pandemic situation. In the online study 2, 86.21% of the women and men reported to be considerably stressed by the pandemic situation.

### Relationships between oral health impairments, depressive symptoms, eating disorders (EDs), anxiety, affect and perceived stress

#### Online study 1

Correlation analyses revealed a number of significant results in the online study 1 as well as in the online study 2. In the online study 1, significant correlations were found between the OHIP-14 and the BDI-II as well as between the OHIP-14 and the EDI-2 subscales. The sum score of the OHIP-14 was significantly, though moderately, correlated with the BDI sum score, *r* = 0.23; *p* = 0.008. In addition, significant positive correlations were found between the severity of depressive symptoms as assessed with the BDI-II and the OHIP-14 subscales “psychological discomfort”, *r* = 0.21; *p* = 0.017, and “psychological disability”, *r* = 0.37; *p* < 0.000, including a reduced ability to concentrate. These two scales measure impairments in well-being and cognition due to oral problems. The sum score of the OHIP-14 was also significantly correlated with the EDI-2 diagnostic subscale “bulimia”, *r* = 0.19; *p* = 0.027. Moreover, self-reported bulimia symptoms were, akin to depressive symptoms, correlated with the OHIP-14 subscales “psychological discomfort”, *r* = 0.19; *p* = 0.033. In addition, self-reported bulimia symptoms were positively correlated with the OHIP-subscale “social disabilities”, *r* = 0.20; *p* = 0.024, and the EDI-2 subscale “dissatisfaction with one’s body” was positively correlated with the subscale “psychological discomfort”, *r* = 0.18; *p* = 0.041 in the OHIP-14. In addition, the EDI-2 sum score as well as the scores of the subscales drive for thinness and body dissatisfaction were significantly correlated with the BDI-II sum scores, supporting a relationship between depressive symptoms and symptoms related to eating disorders such as body dissatisfaction, *r* = 0.24; *p* = 0.006. Only a trended significant correlation was found for the EDI subscale bulimia and depressive symptoms, *r* = 0.16; *p* = 0.059; the subscale drive for thinness and self-reported depressive symptoms were correlated significantly, *r* = 0.18; *p* = 0.042. In addition, significant correlations were found for some of the OHIP-14 subscales or the total OHIP-14 score and affect as well as trait or state anxiety, respectively (see Table 1). Notably, the correlations between the OHIP-14 and trait or state anxiety were higher than the correlation between the correlation between the OHIP-14 and depression (see Table 1). The correlation matrix of the sample of the online study 1 for oral health, depression, anxiety, affect and risk of eating disorders is shown in Table 1. The participants’ health behavior, self-reported smoking behavior in particular, before the pandemic and as a response to the pandemic, was correlated with self-reported oral health impairments in the OHIP-14 subscale (psychological discomfort, *r* = 0.185, *p* < 0.0001). In addition, regular doctoral visits for health promotion (before and during the pandemic) was inversely correlated with self-reported oral health impairments on the OHIP subscale “functional limitations”,* r* = -0.172, *p* < 0.0001). Awareness and perception of changes in physical and sensory bodily functions were largely uncorrelated with oral health complaints. The only items that showed a significant correlation with the OHIP-14 sum score were the PBC items asking for awareness of changes in body temperature and overall sensitivity towards changes in inner tension, *r* = 0.172 and *r* = 0.187; both* p* < 0.001.

**Table 1:** Correlations (Pearson’s) between oral health impairments, depressive symptoms, eating disorders (EDs), anxiety (trait and state), and affect (positive and negative affect)) of the participant sample (women) of the online study 1

Variables	Affect PA	Affect NA	State ANX	Trait ANX	DEPR
AFFECT PA	**1**	**-0.239**	**-0.476**	**-0.386**	**-0.340**
AFFECT NA	**-0.239**	**1**	**0.800**	**0.577**	**0.576**
STATE ANX	**-0.476**	**0.800**	**1**	**0.685**	**0.638**
TRAIT ANX	**-0.386**	**0.577**	**0.685**	**1**	**0.691**
DEPR	**-0.340**	**0.576**	**0.638**	**0.691**	**1**
OHIP (total)	-0.160	**0.238**	**0.320**	**0.301**	**0.227**
OHIP1	**-0.175**	**0.267**	**0.224**	**0.183**	0.134
OHIP2	-0.072	0.167	**0.171**	**0.197**	0.156
OHIP3	-0.137	**0.291**	**0.386**	**0.296**	**0.207**
OHIP4	**-0.180**	0.141	**0.210**	**0.177**	0.112
OHIP5	-0.150	**0.241**	**0.319**	**0.374**	**0.371**
OHIP5	-0.089	0.069	0.167	**0.173**	0.098
OHIP7	-0.123	0.048	0.161	0.152	0.094
EDs	**-0.172**	**0.275**	**0.328**	**0.391**	**0.234**
THIN	-0.102	**0.255**	**0.268**	**0.328**	**0.176**
BUL	-0.089	**0.333**	**0.265**	**0.313**	0.164
BDISS	**-0.212**	**0.172**	**0.300**	**0.351**	**0.237**
Variables	EDs		Thin	Bul	BDISS
AFFECT PA	**-0.172**		-0.102	-0.089	**-0.212**
AFFECT NA	**0.275**		**0.255**	**0.333**	**0.172**
STATE ANX	**0.328**		**0.268**	**0.265**	**0.300**
TRAIT ANX	**0.391**		**0.328**	**0.313**	**0.351**
DEPR	**0.234**		**0.176**	0.164	**0.237**
OHIP (total)	0.163		0.092	**0.191**	0.154
OHIP1	-0.003		-0.023	0.121	-0.048
OHIP2	0.101		0.052	0.095	0.111
OHIP3	0.156		0.073	0.145	**0.177**
OHIP4	0.126		0.103	**0.185**	0.074
OHIP5	**0.182**		0.112	0.161	**0.190**
OHIP5	0.136		0.095	**0.196**	0.094
OHIP7	0.110		0.053	0.164	0.093
EDs	**1**		**0.919**	**0.688**	**0.888**
THIN	**0.919**		**1**	**0.605**	**0.702**
BUL	**0.688**		**0.605**	**1**	**0.374**
BDISS	**0.888**		**0.702**	**0.374**	**1**

Scores printed in bold indicate significance (two-sided, *p* < .05). Abbreviations: THIN (EDI-2 drive for thinness), BUL (EDI-2 bulimia), and BDISS (EDI-2 body dissatisfaction). OHIP1 (functional limitation), OHIP2 (physical pain), OHIP3 (psychological discomfort), OHIP4 (physical disability), OHIP5 (psychological disability), OHIP6 (social disability), and OHIP7 (handicap). AFFECT PA (PANAS positive affect), AFFECT NA (PANAS negative affect), STATE ANX/TRAIT ANX (STAI state and trait anxiety), DEPR (BDI-II)

#### Online study 2

The results of the correlation analyses of the online study 2 support the results found in study 1 regarding the relationship between self-reported impairments in oral health and self-reported depressive symptoms. The sum score of the OHIP-49 was significantly correlated with the BDI-II sum score, *r* = 0.490; *p* = 0.012. In addition, significant positive correlations were found between the severity of depressive symptoms as assessed with the BDI-II and four of the subscales of the OHIP-49 including the OHIP subscales “functional limitation”, *r* = 0.473 *p* = 0.007, “physical pain”, *r* = 0.413; *p* = 0.026, “psychological discomfort”, *r* = 0.47;* p* = 0.010, and “physical disability”, r = 0.40; *p* = 0.030, respectively. Self-reported perceived stress (SCI sum score) was not significantly correlated with the OHIP-49 sum score nor with the subscales of the OHIP-49 questionnaire, all *p* > 0.05. However, self-reported perceived psychophysiological stress symptoms as assessed with the SCI showed a significant correlation with overall oral health complaints assessed with the OHIP-49 as well as with all subscales of the OHIP-49 (see Table 2). No significant correlations were found between the stress coping strategies as assessed with the SCI and oral health impairments as assessed with the OHIP-49 questionnaire. However, coping of stress by alcohol consumption was significantly correlated with self-reported oral health complaints on the OHIP-49 subscale “social disabilities”, *r* = 0.393, *p* = 0.035. In addition, the self-construed survey items asking specifically for stress-related impairments in oral health in the last months were significantly correlated with the OHIP-49 sum score and all OHIP-49 subscales (all *p* < 0.05). In addition, self-reported severity of daily stress perception, single item, Likert scale 1–7, *r* = 0.0452, *p* < 0.05, as well as paying attention to physical and sensory bodily changes during the pandemic, *r* = 0.368, *p* < 0.005, were positively correlated with oral health complaints as assessed by the OHIP-49 sum score. In addition, correlation analysis were performed for the BDI-II and the SCI subscales, revealing no significant correlations with the sum score of the SCI but with self-reported perceived psychophysiological stress symptoms, *r* = 0.751,* p* < 0.001.

**Table 2:** Correlations (Pearsons r) between oral health impairments, severity of depressive symptoms and perceived stress

Variables	DEPR	Stress body	Stress active	Stress positive	Stress alcohol	Stress support	Stress religion	Perceived stress
OHIP1	**0.490**	**0.460**	-0.001	-0.103	0.357	-0.138	0.107	-0.071
OHIP2	**0.413**	**0.370**	0.200	0.324	0.024	-0.115	0.014	0.205
OHIP3	**0.473**	**0.505**	0.014	0.129	0.133	0.023	0.000	0.085
OHIP4	**0.404**	**0.487**	-0.160	-0.145	0.315	-0.199	0.203	-0.145
OHIP5	0.315	**0.537**	-0.096	0.146	0.237	-0.018	0.017	0.037
OHIP6	0.335	**0.505**	-0.227	-0.138	**0.393**	-0.188	0.152	-0.186
OHIP7	0.363	**0.555**	-0.150	-0.001	0.320	-0.134	0.117	-0.073
OHIP (total)	**0.462**	**0.553**	-0.044	0.063	0.269	-0.126	0.095	-0.001

Scores printed in bold indicate significance (two-sided, *p* < .05). Abbreviations: OHIP1 (functional limitation), OHIP2 (physical pain), OHIP3 (psychological discomfort), OHIP4 (physical disability), OHIP5 (psychological disability), OHIP6 (social disability), and OHIP7 (handicap), DEPR (BDI-II)

### Group Differences: Exploratory Analysis (online study 1 only)

To scrutinize the results of the correlation analyses for the all-female sample, the relationships between self-reported oral health impairments and self-reported depressive symptoms or symptoms of eating disorders were statistically explored by means of group comparisons that compared the group of those participants reporting no oral symptoms (*n* = 55) against the group of participants who reported oral symptoms (*n* = 77). In addition, it was explored whether the participants who reported no depressive symptoms and no eating disorder symptoms in the BDI-II or EDI-2 scores differed significantly in self-reported oral symptoms as assessed by the OHIP-14 sum score from those participants, whose BDI or EDI-2 sum scores were within the range of the normative clinical reference samples (see 2.2 Data Analysis). These confirmatory exploratory group comparisons were performed for the data from the online study 1 only, due to the small sample size of the study 2.

#### Risk of EDs or depressive symptoms in the group of participants (all-female) reporting no oral health related symptoms vs. the group of participants (all-female) reporting oral health impairments

The group of participants (all-female sample) who reported oral health impairments on the OHIP questionnaire differed significantly in depressive symptoms, *F*(1,131) = 13.44,* p* = 0.0036, as well as in state, *F*(1,131) = 12.75, *p* = 0.005, and trait anxiety, *F*(1,131) = 11.24, *p* = 0.001, but not in self-reported symptoms related to eating disorders, all *p* > 0.05, from the group of participants who reported no oral health impairments on the OHIP questionnaire.

#### Oral health impairments in the groups of participants (all-female) reporting no depressive symptoms or no eating disorders (EDs) vs. the groups of participants reporting depressive symptoms or symptoms related to eating disorders

The group of participants who reported depressive symptoms or symptoms of eating disorders did not significantly differ from the group of participants who reported no depressive symptoms and whose scores on the EDI-2 scores fell below that of the clinical reference samples (see 2.2 Data Analysis). Only the participants with subclinical scores on the EDI-2 subscale of body dissatisfaction marginally differed in self-reported overall oral health impairments on the OHIP from the participants with low scores on the body dissatisfaction scale of the EDI-2 questionnaire, *F*(1,131) = 3.428, *p* = 0.066.

## Discussion

Oral diseases and mental disorders are major health burdens affecting people irrespective of age. Recently, the assumption of a relationship between oral health and mental health has been discussed with rising attention and proof in the literature (e.g., Kisely, 2016). So far, the focus has been on the investigation of vulnerable target groups who already suffer from mental disorders such as depressive disorders or eating disorders (see Introduction). However, more recent studies conducted in samples of young healthy adults in several countries point to an interesting paradox of better subjective oral health in the elderly compared with young or middle-aged adults (Hagman et al., 2021). Given the prominence and increasing prevalence of depression and eating disorders in the general population, particularly in the population of young adults, the present studies thought to answer whether oral health impairments and mental health conditions, predominantly related to depressive symptoms and symptoms related to eating disorders are already linked in healthy young adults without clinical diagnosis of depression or eating disorders and without full blown oral diseases such as tooth loss, caries or periodontitis. In line with answering this question, several psychological factors were assessed and considered in the two studies reported in this manuscript to elucidate what factors could be potential risk factors promoting the relationship between oral and mental symptoms. In particular, the hypotheses were tested that psychological stress, most notably the individually perceived stress level, related stress coping strategies, one’s health behavior, trait and state anxiety, and individual differences in the sensitivity for changes in physical and sensory bodily functions could influence oral health and mental health, respectively. The results of the two online studies comprising an all-female sample, *N* = 133, as well as a mixed gender sample of smaller sample size, *N* = 29, support the above summarized assumptions. Self-reported impairments in oral health as assessed with the short and the long version of the OHIP questionnaire (John et al., 2002) and self-reported symptoms of depression, trait and state anxiety as well as of eating disorders as assessed with standardized questionnaires including the BDI-II (German version: Hautzinger 2010), STAI (German version: Laux et al., 1981) and EDI-2 (German version: Thiel & Paul, 2006) subscales body dissatisfaction, bulimia, and drive for thinness, were significantly and positively correlated in the all-female sample of the online study 1. The correlations between self-reported impairments in oral health and self-reported depressive symptoms were also found in study 2 encompassing the smaller sample of women and men. The correlations show that links between self-reported impairments in oral health and in mental health can occur together in healthy participant samples who so far report no history or current diagnosis of mental disorders nor any significant oral diseases such as tooth loss. Interestingly, the additional group statistics of the all-female sample that compared the participants reporting no oral health impairments vs. the group of participants reporting oral health impairments in the OHIP-14 questionnaire suggest a significant difference in depressive symptoms, anxiety symptoms and eating disorder symptoms as a function of group (no self-reported oral health symptoms vs. self-reported oral health symptoms). In contrast, the comparisons between the groups reporting no symptoms vs. depressive or eating disorder symptoms did not differ in self-reported oral health impairments. In the all-female sample of the online study 1 and in the mixed gender sample of the online study 2, self-reported depressive symptoms were evident in a number of participants as were oral health complaints. On average, 65.41% of the participants of the online study 1 (all-female) and 79.31% of the participants of the online study 2 (mixed gender sample) reported no depressive symptoms during the time period of the study assessment, whereas 15.8% of the participants of the online study 1 (all-female) and 6.90% of the online study 2 (mixed gender sample) had a depression score indicating subclinical depression during the time period of the study assessment (cut off score of 14 and higher). In addition, the self-reported scores on the EDI-2 indicated a risk of eating disorders in the all-female sample of the online study 1 in 15.79% (drive for thinness), 18.18% (body dissatisfaction) and 31.58% (bulimia) of the participants. In the all-female sample only 55.6% of the participants did not report any or little impairments in oral health; in the online study 2, only 27.59% of the participants reported no or little impairments in oral health. Taken together, this suggest that a risk of depression or a risk of eating disorders in healthy adults are no sufficient preconditions for reporting impairments in oral health, whereas self-reported oral health impairments seem to significantly discriminate between individuals reporting depressive symptoms or symptoms of eating disorders. In other words, this suggests that in yet healthy samples of young adults, oral health complaints could be reliable predictors of the risk of depression and the risk of eating disorders but that within the group of individuals at risk of depression or at risk of eating disorders not all individuals are suffering from oral health impairments. Of course this causal interpretation is still speculative, was tested in the all-female sample only and therefore requires validation in future studies with larger sample sizes and mixed gender samples. Moreover, a replication would be helpful to scrutinize the additional results obtained in the present study that suggest that potentially relevant psychological variables such as anxiety, stress or one’s health behavior including individual differences in the sensitivity and awareness to bodily functions could play a role in influencing the degree of self-reported oral health complaints. Previous studies investigating the relationship between depressive and eating disorders and oral diseases in clinical groups suggest that in clinical samples the relationship between mental health conditions, oral diseases and impairments in oral health can be explained by psychological factors such as reduced oral hygiene behavior, loss of motivation, medication intake or detrimental nutrition and eating behavior supporting erosion of teeth and gum (Frydrych et al., 2005; Kisely et al., 2015; Kisely et al., 2016). In the present studies, medication intake (psychotropic drugs) was an exclusion criterion and overall health behavior as assessed via the survey studies to additionally control for behavior change provoked by the COVID-19 pandemic including oral hygiene behavior was on average well pronounced in the study samples. However, due to the pandemic lockdowns individuals reported to have reduced their regular doctor visits including those by the dentist. In addition, in the online study 2, stress coping by means of alcohol consumption was positively correlated with self-reported oral symptoms and smoking behavior (albeit only little pronounced in the present study sample) was positively correlated with oral health impairments pre- and during the Covid-19 pandemic. Similarly, overall body awareness, i.e., the overall sensitivity towards bodily changes as assessed with items from the PBC scale (Miller et al., 1981) was not correlated with self-reported oral health impairments in the online study 1 but a correlation with oral health impairments was found with single PBC items measuring the sensitivity to certain physical changes such as temperature or dryness of mouth. These relations were partially replicated in the online study 2 using self-construed items. Smoking and alcohol consumption are lifestyle related risk factors involved in a number of health impairments. Similarly, body awareness has been discussed as a factor contributing positively or negatively to mental health concerns (for an overview Mehling et al., 2009). In particular in individuals with anxiety proneness a heightened body awareness has been suggested to support the experience of negative bodily changes (for an overview Mehling et al., 2009). The results of the present study confirm that in the all-female sample of the online study 1, trait and state anxiety were positively correlated with oral health impairments and with depressive symptoms as well as with body dissatisfaction and drive for thinness and marginally with bulimia (as symptoms of eating disorders). The results support a role of anxiety in the emergence of oral health complaints and in the emergence of depression and eating disorders. Similarly, the results of the online study 2 suggest that stress but not overall stress coping seems to be linked to oral health complaints and the severity of self-reported health symptoms in healthy subjects (e.g., see also Vasiliou et al., 2016). Crucially, two dimensions of stress turned out to correlate significantly with self-reported oral health complaints and with depressive symptoms, i.e., the perception of psychophysiological stress symptoms (as measured by standardized questionnaires) and the experience of suffering from daily stressors as measured by single survey items. Furthermore, specific items asking for feeling stress by oral health complaints including the perception of stress-related changes in taste and smelling were significantly correlated with self-reported oral symptoms assessed with the OHIP-49 questionnaire and its subscales in the online study 2. Theoretically, stress and its facets could influence oral health and mental health and its potential relationship as follows: on one hand, perceived stress could be a mediator: i.e., perceived stress correlates with oral health as well as with mental health impairments. On the other hand, it could be, that stress specifically impairs oral health or mental health or vice versa. The present results suggest that in healthy young adults, the health effects of perceived stress, especially of the perception of psychophysiological stress symptoms and the experience of feeling stressed on a daily level, are not disease specific but contributing to both, the experience of oral and mental symptoms (depressive symptoms), possibly by enhancing stress-related physical symptoms affecting musculoskeletal, respiratory, cardiovascular, endocrine, gastrointestinal, and the nervous system, especially when stress symptoms are experienced daily. In line with this suggestion, the subscale psychophysiological stress symptoms of the SCI questionnaire (Satow, 2012) as well daily stress (single survey item) were significantly correlated with self-reported oral complaints assessed with the OHIP questionnaire (John et al., 2002) as well as with self-reported depressive symptoms as assessed with the BDI-II (Hautzinger 2010). Given that assessment was done during the COVID-19 lockdowns it is likely that the pandemic situation intensified the relationships between mental, stress and oral complaints and this already in young still healthy adults.

## Limitations and future research

Taken together, the results reported in this manuscript support a relationship between oral health and mental health in young adults and show that not only in the elderly but additionally in young adults impairments in oral health correlate with symptoms of mental health problems prior to the onset of a clinically manifest disorder. Specifically, the results show that in healthy adults self-reported impairments in oral health can co-vary with the experience of depressive symptoms, with trait as well as state anxiety, negative affect and eating-disorder relevant symptoms assessed by standardized scales measuring disorder-relevant symptoms and subjective concerns related to depression or in case of eating disorders with concerns and preoccupation with weight gain and dieting, preoccupation with weight, fear of weight gain, episodes of binge eating and purging and dissatisfaction with one's physical appearance. The correlations were found despite the participants reporting overall good health behavior and no significant diagnosed oral diseases. Given that the standardized questionnaires used in the present studies capture oral symptoms and most of the mental health symptoms (depression, state anxiety, affect) over a short period of time (e.g., at the moment, 14 days up to a month), it would be interesting to include follow-up assessments in future studies to scrutinize causal relationships. Likewise, the influence of stress on oral health should be scrutinized in future studies. Given that particularly the experience of psychophysiological stress symptoms was correlated with oral health symptoms, experimental laboratory studies are warranted to determine the possible psychophysiological and biopsychological pathways by which psychological stressors affect oral functions such a chewing, taste perception or oral pain perception and how this is affecting well-being and quality of life in healthy adults in the long run and potentially contributing to the experience of symptoms known from mood disorders and eating disorders. Although the present studies revealed several important relationships between oral and metal health impairments, the studies do not come without limitations. First, the results are limited to an all-female sample and a mixed-gender sample of very small sample size with both study samples sharing the same socio-educational background and status. Therefore, the results are preliminary and cannot be generalized to a broader population sample with different socio-educational or socioeconomic status. Replication with larger sample sizes including mixed gender samples with different sociodemographic or even cultural background should be conducted to confirm the present relationships between mental and oral health among healthy adults. Second, the two online studies were using on the one hand the same and on the other hand different questionnaires and survey items for psychological assessment. Therefore, the results obtained from the two online studies have been analysed separately and therefore the results only partly complement each other. Future studies could replicate the present findings using the same questionnaire and survey item packages in a community-based population sample to complement recent surveys conducted with non-standardized questionnaires (e.g., Tiwari et al., 2021) to confirm associations between mental health status, oral health status, and oral healthcare utilization as well as changes therein during the COVID-19 pandemic. As mentioned above, future studies should additionally aim at combining self-report measures with more objective measures assessing in addition to self-reported oral health complaints the dental status and the functional status of the oral cavity in addition to self-reported mental health complaints and the psychophysiological correlates thereof. Moreover, from a theoretical point of view, future studies should go beyond exploratory testing of hypotheses and test specific models that explain the relationship between oral and mental health theoretically (e.g., see Halvari et al., 2013).

## Supplementary Information

Below is the link to the electronic supplementary material.

Supplementary file1 (DOCX 44.2 KB)

## Data Availability

The datasets generated during and/or analysed during the current study are not publicly available because in the informed consent form the possibility of raw data being published online was not explicitly stated to the participants or approved by the participants but the group-level data as it is provided in this manuscript are available from the corresponding author on reasonable request.
