# Supplementary material for: Oral health and mental health in healthy adults, a topic of primary prevention and health care, empirical results from two online studies
Source: Curr Psychol. 2023 Jan 7:1–15. Online ahead of print. doi: 10.1007/s12144-022-04121-8 (PMC9825076; doi:10.1007/s12144-022-04121-8)
Supplement: Supplementary file 1 — Supplementary file1 (DOCX 44.2 KB) [file 12144_2022_4121_MOESM1_ESM.docx]

**Oral health and mental health in healthy adults, a topic of primary**

**prevention and health care, empirical results from two online studies**

**Cornelia Herbert**

**SUPPLEMENT**

**Table 1A** Overview of the survey items and questionnaires included in the studies (study 1 or study 2)

| **Domain** | **questionnaire** | **subscales** | |
| --- | --- | --- | --- |
| Sociodemographic questions | Open/closed questions | sex, age, nationality, marital status, education level, job, studies, language skills, diseases, medication, handedness, height, weight |  |
|  | Beck Depression Inventory (BDI-II), German version, study 1 and study 2 | Depression items  (except suicide item) |  |
| Standardized questionnaires | Eating Disorder Inventory (EDI-2), German short version, study 1  Spielberger State and Trait Inventory (STAI), German version,  study 1  Positive Affect and Negative Affect Scales (PANAS), German version,  study 1 and study 2 | Drive for thinness, bulimia, body dissatisfaction  State anxiety scale and trait anxiety scale  Positive affect scale and negative affect scale |  |
|  | Oral health impact profile (OHIP) German full version/ German short version  study 1 (OHIP-14)  study 2 (OHIP-49) | Seven subscales: functional limitation, physical pain, psychological discomfort, physical disability, psychological disability, social disability, handicap |  |
|  | Stress Coping Inventory (SCI)  study 2 | Stress due to uncertainty, stress due to excessive demands, stress due to experience of negative life events, total stress  subscales: psychosomatic stress symptoms, stress coping strategies, positive thinking, active coping, social support seeking, belief/religion, alcohol and drug consumption |  |
| Body Perception/Awareness | Body Perception Questionnaire Body Awareness Very Short Form, German translation  study 1  Self-construed items  study 2 | Changes in body temperature, stomach, heart, mouth and throat, body tension  Changes in taste, smell, cardiac activity, mouth (during the assessment period /Covid-19 pandemic) |  |
|  |  |  |  |
| Items  Health Behavior | Self-construed items according to Multiple Health Behavior  study 1 and study 2  Self-construed items – oral sensitivity  study 2 only | Exercise, recreation, smoking, stress regulation, sleep, alcohol, healthy nutrition, nature, doctor visits,  paying attention to chewing, controlling what to eat, regular oral hygiene and dental visits |  |
|  |  |  |  |
| Pandemic questions | Self-construed items  study 1 and study 2 | Job, working hours, well-being, mood, health behavior, sensations (taste, smell, body), etc. |  |
| Severity of daily stressors  Stress-related oral health complaints (last month) | Self-construed single items  study 1 and study 2  Self-construed items, study 2 | Perception of overall daily stressors, stress-related symptoms regarding teeth and mouth |  |

**Table 2A** Overview of the average scores of the all-female sample of the online study 1 (n=133) including age and self-report questionnaires: Abbreviations: BMI (body mass index), P-AFFECT (PANAS positive affect), N-AFFECT (PANAS negative affect), STATE ANX/TRAIT ANX (STAI state and trait anxiety), DEPRESSION (BDI-2), THIN (EDI-2 drive for thinness), BUL (EDI-2 bulimia), and BDISS (EDI-2 body dissatisfaction). OHIP1 (functional limitation), OHIP2 (physical pain), OHIP3 (psychological discomfort), OHIP4 (physical disability), OHIP5 (psychological disability), OHIP6 (social disability), and OHIP7 (handicap), SD (Standard Deviation).

| **Variable** | **N** | | **Minimum** | **Maximum** | **% Minimum** | **% Maximum** | **Mean** | **SD** |
| --- | --- | --- | --- | --- | --- | --- | --- | --- |
| Age | | 133 | 18 | 65 | 3 | 1 | 25.02 | 7.92 |
| BMI | 133 | | 16 | 39.64 | 1 | 1 | 21.81 | 3.66 |
| P-AFFECT | 133 | | 13 | 44 | 1 | 2 | 28.21 | 6.69 |
| N-AFFECT | 133 | | 10 | 32 | 33 | 2 | 13.70 | 4.56 |
| STATE ANX | 133 | | 21 | 78 | 1 | 1 | 37.42 | 10.41 |
| TRAIT ANX | 133 | | 20 | 72 | 1 | 2 | 38.29 | 10.68 |
| DEPRESSION | 133 | | 0 | 40 | 5 | 1 | 7.89 | 7.32 |
| OHIP (total) | 133 | | 0 | 40 | 55 | 1 | 2.76 | 4.70 |
| OHIP1 | 133 | | 0 | 2 | 119 | 3 | 0.13 | 0.40 |
| OHIP2 | 133 | | 0 | 8 | 72 | 1 | 0.89 | 1.34 |
| OHIP3 | 133 | | 0 | 8 | 82 | 1 | 0.86 | 1.44 |
| OHIP4 | 133 | | 0 | 6 | 124 | 1 | 0.14 | 0.63 |
| OHIP5 | 133 | | 0 | 3 | 100 | 5 | 0.41 | 0.80 |
| OHIP6 | 133 | | 0 | 8 | 119 | 1 | 0.18 | 0.79 |
| OHIP7 | 133 | | 0 | 7 | 121 | 1 | 0.15 | 0.69 |
| EDI-2 | 133 | | 28 | 119 | 1 | 1 | 56.85 | 18.86 |
| THIN | 133 | | 7 | 42 | 7 | 1 | 17.79 | 7.82 |
| BUL | 133 | | 7 | 29 | 9 | 1 | 12.59 | 4.68 |
| BDISS | 133 | | 9 | 51 | 1 | 1 | 26.47 | 9.52 |

**Table 3A** Overview of the participants’ self-reported health behavior in the all-female sample of the online study 1 (n=133). Health behavior items included exercising/sports, recreation/relaxation, smoking, stress regulation (avoiding stressful situations and anger), sleep, alcohol consumption, healthy food and nutrition, nature (physical activity during leisure time), and regular visits to the doctor for health prevention. Scales for each item ranged from 0 (never) to 5 (almost always).

| Health Behavior  (before the pandemic) | Reponse | % | | Health Behavior  (during the pandemic) | | % | |
| --- | --- | --- | --- | --- | --- | --- | --- |
| Exercise/sports | never | 6.49 | Exercise/sports | | 12.99 | |  |
|  | barely | 14.29 |  | | 16.88 | |  |
|  | sometimes | 19.48 |  | | 22.08 | |  |
|  | often | 45.45 |  | | 27.27 | |  |
|  | almost always | 14.29 |  | | 20.78 | |  |
| Relaxation/Recreation | never | 6.49 | Relaxation | | 3.90 | |  |
|  | barely | 15.58 |  | | 14.29 | |  |
|  | sometimes | 29.87 |  | | 25.97 | |  |
|  | often | 38.96 |  | | 33.77 | |  |
|  | almost always | 9.09 |  | | 22.08 | |  |
| Smoking | never | 85.71 | Smoking | | 89.61 | |  |
|  | barely | 5.19 |  | | 2.60 | |  |
|  | sometimes |  |  | |  | |  |
|  | often | 5.19 |  | | 5.19 | |  |
|  | almost always | 3.90 |  | | 2.60 | |  |
| Avoiding feeling stressed and angry | never | 1.30 | Avoiding feeling stressed and angry | | 2.60 | |  |
|  | barely | 15.58 |  | | 11.69 | |  |
|  | sometimes | 19.48 |  | | 19.48 | |  |
|  | often | 46.75 |  | | 46.75 | |  |
|  | almost always | 16.88 |  | | 19.48 | |  |
| Enough sleep | never | 1.30 | Enough sleep | | 5.19 | |  |
|  | barely | 5.19 |  | | 0.00 | |  |
|  | sometimes | 15.58 |  | | 9.09 | |  |
|  | often | 38.96 |  | | 27.27 | |  |
|  | almost always | 38.96 |  | | 58.44 | |  |
| Alcohol consumption | never | 19.48 | Alcohol consumption | | 36.36 | |  |
|  | barely | 38.96 |  | | 32.47 | |  |
|  | sometimes | 0.00 |  | | 0.00 | |  |
|  | often | 33.77 |  | | 25.97 | |  |
|  | almost always | 7.79 |  | | 5.19 | |  |
| Healthy eating | never | 1.30 | Healthy eating | | 2.60 | |  |
|  | barely | 6.49 |  | | 6.49 | |  |
|  | sometimes | 22.08 |  | | 16.88 | |  |
|  | often | 45.45 |  | | 42.86 | |  |
|  | almost always | 24.68 |  | | 31.17 | |  |
| Physical Activity during leisure time | never | 2.60 | Physical Activity during leisure time | | 6.49 | |  |
|  | barely | 19.48 |  | | 7.79 | |  |
|  | sometimes | 28.57 |  | | 14.29 | |  |
|  | often | 33.77 |  | | 42.86 | |  |
|  | almost always | 15.58 |  | | 28.57 | |  |
| Regular doctor visits | never | 12.99 | Regular doctor visits | | 44.16 | |  |
|  | barely | 20.78 |  | | 16.88 | |  |
|  | sometimes | 18.18 |  | | 7.79 | |  |
|  | often | 22.08 |  | | 12.99 | |  |
|  | almost always | 25.97 | |  | 18.18 | |  |

**Table 3B** Overview of the participants’ self-reported health behavior and body awareness in the mixed gender sample of the online study 2 (n=29). Health behavior items included exercising/sports, recreation/relaxation, smoking, stress regulation (avoiding stressful situations and anger), sleep, alcohol consumption, healthy food and nutrition, nature (physical activity during leisure time), and regular visits to the doctor/dentist for health prevention. Body awareness included paying items asking for paying attention to bodily changes related to e.g., breathing, muscle tension, cardiac activity, taste or smell. Scales for each item ranged from 0 (never) to 5 (almost always).

| **Health Behavior** | | **Response** | **%** |
| --- | --- | --- | --- |
| Exercise/sports | | almost always | 20.69 |
|  | | sometimes | 27.59 |
|  | | often | 41.38 |
|  | | barely | 10.34 |
| Relaxation/Recreation | | sometimes | 37.93 |
|  | | often | 48.28 |
|  | | barely | 13.79 |
| Smoking | | never | 89.66 |
|  | | often | 3.45 |
|  | | barely | 6.90 |
| Avoiding feeling stressed and angry | | sometimes | 37.93 |
|  | | often | 51.72 |
|  | | barely | 10.34 |
| Enough sleep | | almost always | 34.48 |
|  | | sometimes | 13.79 |
|  | | never | 3.45 |
|  | | often | 44.83 |
|  | | barely | 3.45 |
| Alcohol consumption | | sometimes | 27.59 |
|  | | never | 31.03 |
|  | | often | 10.34 |
|  | | barely | 31.03 |
| Healthy eating | | almost always | 34.48 |
|  | | often | 58.62 |
|  | | barely | 6.90 |
| Physical Activity (during leisure time) | | almost always | 20.69 |
|  | | sometimes | 24.14 |
|  | | often | 48.28 |
|  | | barely | 6.90 |
| Regular doctor visits | | almost always | 48.28 |
|  | | sometimes | 13.79 |
|  | | often | 27.59 |
|  | | barely | 10.34 |
| Regular visits to the dentist | | almost always | 75.86 |
|  | often | | 6.90 |
|  | sometimes | | 10.35 |
|  | barely | | 3.45 |

never 3.45

| Regular daily tooth brushing (2times/day) | almost always | 79.31 |  |
| --- | --- | --- | --- |
|  | often | 10.35 |  |
|  | sometimes | 3.45 |  |
|  | barely | 3.45 |  |
|  | never | 3.45 |  |
| Body Awareness/Perception  Paying attention to physical changes (heart, taste, smell, …) | almost always | 6.90 |  |
|  | often | 13.80 |  |
|  | sometimes  barely  never | 13.80  27.59  10.35 |  |

**Table 4A** Overview of the oral health average scores of the sample of online study 2 (n=29). OHIP1 (functional limitation), OHIP2 (physical pain), OHIP3 (psychological discomfort), OHIP4 (physical disability), OHIP5 (psychological disability), OHIP6 (social disability), and OHIP7 (handicap), and OHIP total score (all scales).

| **Oral Health** | **Mean** | **SD** |
| --- | --- | --- |
| OHIP1 | 2.66 | 3.48 |
| OHIP2 | 3.83 | 5.55 |
| OHIP3 | 2.21 | 3.59 |
| OHIP4 | 1.48 | 4.41 |
| OHIP5 | 1.72 | 3.85 |
| OHIP6 | 0.83 | 2.45 |
| OHIP7 | 1.52 | 3.77 |
| OHIP | 14.24 | 23.52 |
